# Enhanced Anti-Lung Cancer Effects of Steamed *Panacis Japonici Rhizoma*: Insights from Metabolomics, Network Pharmacology and Molecular Dynamics Simulation

**DOI:** 10.3390/ijms262411999

**Published:** 2025-12-13

**Authors:** Yijia Zhang, Jingxiao Yang, Binqing Qu, Jiacheng Huang, Yuanqing Wang, Jianye Yan

**Affiliations:** 1 Academy of Chinese Medical Sciences (Science and Technology Innovation Center), Hunan University of Chinese Medicine, Changsha 410208, China; 20243829@stu.hnucm.edu.cn (Y.Z.); yangjingxiao@stu.hnucm.edu.cn (J.Y.); qubinqing@stu.hnucm.edu.cn (B.Q.); jiacheng.huang@stu.hnucm.edu.cn (J.H.); 2 College of Life Science and Technology, Central South University of Forestry and Techology, Changsha 410004, China; 3 Hunan Engineering Technology Research Center for Bioactive Substance Discovery of Chinese Medicine, Changsha 410208, China; 4 Hunan Province Sino-US International Joint Research Center for Therapeutic Drugs of Senile Degenerative Diseases, Changsha 410208, China

**Keywords:** *Panacis Japonici Rhizoma*, metabolomics, network pharmacology, molecular docking, molecular dynamics simulation, lung cancer

## Abstract

*Panacis Japonici Rhizoma* (PJR), a medicinal and edible herb of the *Panax* genus, exhibits enhanced anti-lung cancer activity after steaming, a phenomenon consistent with other *Panax* species. However, the active constituents responsible for this improved efficacy and their underlying mechanisms remain unclear. In this study, we integrated UPLC-Q-TOF-MS–based metabolomics, network pharmacology, molecular docking, molecular dynamics simulations, and in vitro assays to identify the key metabolites and elucidate the mechanistic basis of steamed PJR against lung cancer. Metabolomic analysis revealed ten significantly upregulated metabolites following steaming. Network pharmacology analysis identified AKT1, EGFR, HSP90AA1, SRC, and STAT3 as core targets, primarily enriched in the MAPK, PI3K-Akt, and Ras signaling pathways. Molecular docking and molecular dynamics simulations further demonstrated stable interactions between major metabolites and core targets. In vitro experiments confirmed that steamed PJR exerted markedly stronger anti-tumor effects than its raw form. Collectively, these findings indicate that steamed PJR acts through a multi-target, multi-pathway mechanism mediated by multiple bioactive constituents, highlighting its therapeutic potential in lung cancer treatment.

## 1. Introduction

*Panacis Japonici Rhizoma* (PJR), the dried rhizome of *Panax japonicus* C.A.Mey (Araliaceae), is a rare, endangered, and valuable medicinal and edible herb. In several southwestern provinces of China, including Guizhou, Sichuan, and Yunnan, PJR is commonly consumed in stews, soups, and medicinal wines for health maintenance. It exhibits tonic and hematopoietic effects comparable to those of *Panax ginseng* and *P. notoginseng* [1]. According to the 2020 edition of the *Pharmacopoeia of the People’s Republic of China*, PJR is effective in dissipating blood stasis, reducing swelling, promoting hemostasis, and tonifying deficiency [2]. Traditionally, it has been used to treat conditions such as hemoptysis associated with consumptive cough, traumatic injuries, phlegm-induced cough, and post-illness debility [3]. PJR is rich in saponins and polysaccharides, with saponins considered their principal bioactive constituents [4]. Modern pharmacological studies have revealed a wide range of biological activities attributable to these components, including anti-aging [5], anti-osteoporosis [6], hypoglycemic [7], anti-tumor [8,9], anti-rheumatic [10], anti-oxidant [11], anti-inflammatory [12], and anti-obesity effects [13].

Lung cancer remains one of the most prevalent malignancies worldwide, with both incidence and mortality continuing to rise, and curative therapies still largely lacking. According to the 2022 Global Cancer Statistics released by the International Agency for Research on Cancer (IARC), lung cancer ranks first globally in both incidence and mortality, accounting for approximately 2.5 million new cases (12.4% of all cancer diagnoses) and 1.8 million deaths (18.7% of all cancer-related mortality) [14]. Among various types of cancer, lung cancer remains a leading cause of cancer-related mortality worldwide, highlighting the need for preventive and adjuvant dietary strategies. Recent studies have demonstrated that saponins extracted from PJR inhibit tumor growth in rat models of lung cancer by modulating the TLR4/NF-κB signaling pathway and suppress the proliferation, migration, and invasion of lung cancer cells via regulation of the PTEN-PI3K-AKT signaling pathway [15,16]. Additionally, PJR saponins have been shown to alleviate cisplatin resistance in lung cancer cells by downregulating the expression of proteins such as MDR1, P-gp, and p-Akt [17].

Herbal processing, a pivotal pharmaceutical intervention in traditional Chinese medicine (TCM), can modify herbal properties, reduce toxicity, enhance therapeutic efficacy, etc. [18]. Extensive research has been conducted on the processing methods of *Panax* species, such as *Panax ginseng*, processed into white, red, and black ginseng [19], and *Panax notoginseng* [20]. Processed *Panax* herbs typically exhibit higher levels of rare saponins and stronger pharmacological activities compared with their raw counterparts [21,22,23]. Rare ginsenosides such as Rg1 and Rb2 have demonstrated prominent concentration-dependent inhibitory effects on the proliferation of specific cancer cells [24,25]; additionally, ginsenosides Rg3, Rh2, and Rg5 exhibit antimicrobial activity [26], and Rg1, Rg3, and Rb1 show anti-diabetic effects [27].

Nevertheless, systematic studies examining compositional and pharmacological differences between raw and steamed forms of PJR, another *Panax* species rich in saponins, remain limited. Given that steaming *Panax ginseng* or *Panax notoginseng* leads to an increase in rare saponins and subsequently enhances anti-tumor activity, we hypothesized that steaming may similarly potentiate the anti-lung cancer efficacy of PJR. Metabolomics, a high-throughput analytical approach enabling comprehensive and unbiased profiling of metabolites in TCM, has been increasingly applied to identify markers of chemical transformations induced by herbal processing [28,29,30]. Integrating metabolomics with multivariate statistical analyses facilitates systematic investigation of the chemical changes occurring during PJR processing and contributes to elucidating its processing mechanism.

Network pharmacology is an innovative approach that integrates big data analytics with systems biology and network biology to comprehensively characterize drug actions within complex biological networks. In the context of TCM, it enables elucidation of the network relationships between multi-component herbal formulations and their multi-target interactions, facilitates the screening of bioactive constituents, and provides mechanistic insights into therapeutic effects through pathway-network mapping [31].

In this study, UPLC-Q-TOF-MS–based comparative metabolomics was employed to characterize the differential metabolites between raw and steamed PJR, which may be associated with its enhanced anti-tumor activity. Network pharmacology was subsequently applied to predict the targets and signaling pathways involved in the anti-lung cancer effects of these metabolites. The predicted anti-lung cancer targets were validated through molecular docking, and molecular dynamics (MD) simulations were performed to further assess the stability of key metabolite-target complexes, thereby strengthening the docking results. Finally, cellular assays were conducted to experimentally confirm the anti-lung cancer efficacy of steamed PJR. By integrating metabolomics, network pharmacology, molecular docking, MD simulations, and in vitro validation, this study aimed to establish a strategy for screening anti-lung cancer constituents in steamed PJR and elucidating the mechanisms underlying the biological activity of its differential metabolites. The findings provide a scientific foundation for the development of functional foods or health products derived from steamed PJR with potential anti-lung cancer benefits.

## 2. Results

### 2.1. Chemical Profiling and Multivariate Statistical Analysis of Distinctive Markers Between Raw and Steamed PJR

**Figure 1:**
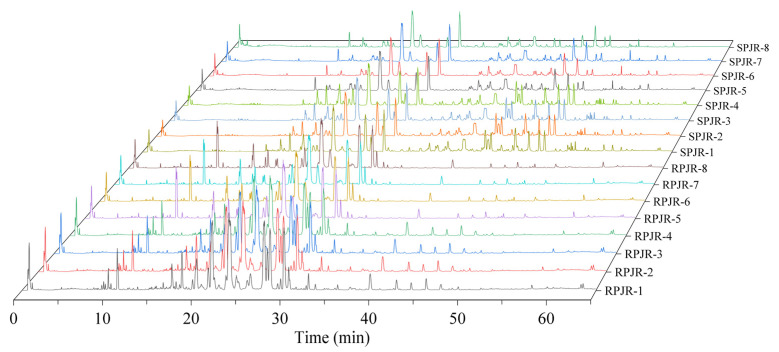
Three-dimensional total ion flow diagram of raw PJR (RPJR) and steamed PJR (SPJR).

**Figure 2:**
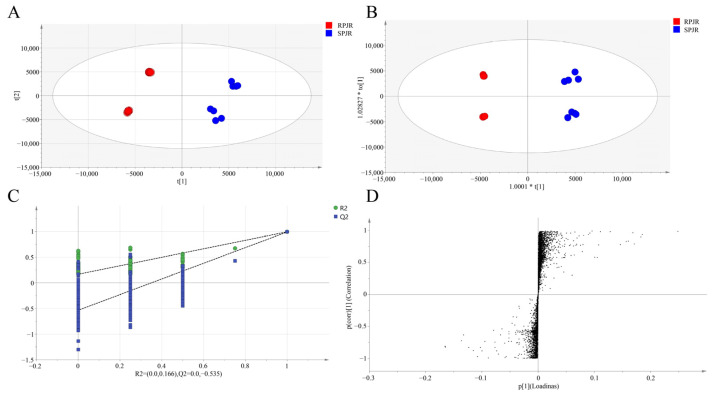
Analysis of metabolites of raw PJR (RPJR) and steamed PJR (SPJR). (**A**) PCA score (R^2^X = 0.967, Q^2^ = 0.898). (**B**) OPLS-DA score (R^2^X = 0.798, R^2^Y = 0.798, Q^2^ = 0.992). (**C**) Permutation test plot (200 permutations). (**D**) S-plot.

The total ion current (TIC) chromatograms of raw and steamed PJR in negative ion mode are illustrated in Figure 1, with detailed peak lists and abundance data provided in Table S1. Principal component analysis (PCA) was performed to visualize the metabolic differences between raw and steamed samples (Figure 2A). The PCA score plot revealed clear separation of the two groups, indicating substantial compositional changes induced by steaming. The model parameters (R^2^X = 0.967, Q^2^ = 0.898) validated the model’s reliability in distinguishing metabolic variations.

To further identify differential metabolites, orthogonal partial least squares-discriminant analysis (OPLS-DA) was conducted (Figure 2B). The OPLS-DA score plot demonstrated significant intergroup separation, consistent with the PCA results. The model parameters were R^2^X = 0.798, R^2^Y = 0.798, and Q^2^ = 0.992. Permutation testing (200 iterations) yielded a Q^2^ regression line with an intercept below zero (Figure 2C), confirming the robustness of the model without overfitting.

In the loading plot (Figure 2D), metabolites located in the upper-right or lower-left quadrants exhibited greater differential significance, based on covariance and correlation coefficients. Biomarker screening was performed by integrating variable importance in projection (VIP) values (VIP > 1) and *t*-test results (*p* < 0.05). As summarized in Table 1, a total of 22 differential metabolites were identified. Compared with raw samples, the upregulated metabolites in steamed PJR included Ginsenoside F1, Ginsenoside F4, Ginsenoside Rg6, Momordin I, (3b,21b)-12-oleanene-3,21,28-triol 28-[arabinosyl-(1→3)-arabinosyl-(1→3)-arabinoside], Ginsenoside Rg3, Vinaginsenoside R1, Momordin Ia, Ginsenoside Rg5, and Ginsenoside Rh2. Conversely, the downregulated metabolites comprised Notoginsenoside J, Notoginsenoside R1, Ginsenoside B2, Notoginsenoside R4, Notoginsenoside Fa, Vinaginsenoside R7, Ginsenoside Rd, Ginsenoside Rs2, Ginsenoside Rs1, Gypenoside XVII, Calenduloside H methyl ester, and Calenduloside G methyl ester.

**Figure 3:**
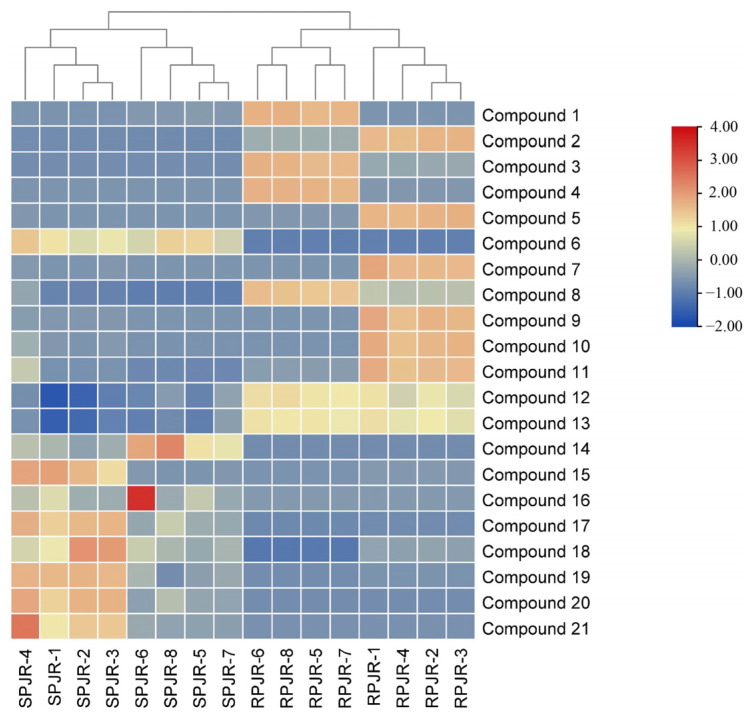
Heat map of 21 metabolites of raw PJR (RPJR) and steamed PJR (SPJR). The color scale ranges from blue (low relative abundance) to red (high relative abundance).

**Figure 4:**
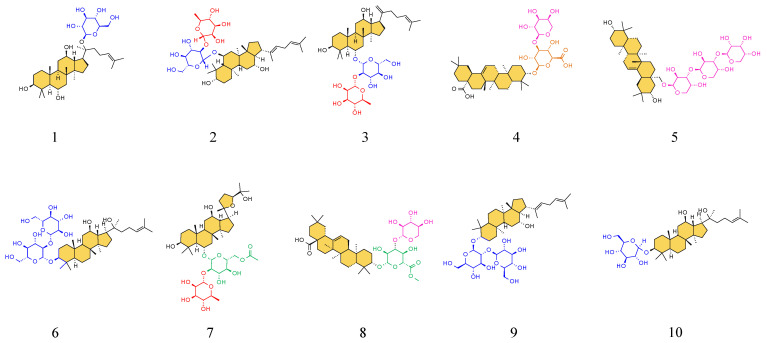
Structures of the ten upregulated compounds. The serial numbers from 1 to 10 are Ginsenoside F1, Ginsenoside F4, Ginsenoside Rg6, Momordin I, (3b,21b)-12-Oleanene-3,21,28-triol 28-[arabinosyl-(1→3)-arabinosyl-(1→3)-arabinoside], Ginsenoside Rg3, Vinaginsenoside R1, Momordin Ia, Ginsenoside Rg5, and Ginsenoside Rh2 in sequence. The saponin aglycone is filled with orange, and the different glycosyl groups are depicted in blue, red, pink, orange, and green.

To further visualize compositional changes, a heat map of the identified metabolites was generated using TBtools 2.120 (Figure 3). The heat map clearly distinguished raw and steamed PJR samples into two separate clusters, consistent with the PCA results. Increased and decreased metabolites in steamed PJR were sharply contrasted. As shown in Figure 4, the significantly upregulated metabolites in steamed samples were Ginsenoside F1, Ginsenoside F4, Ginsenoside Rg6, Momordin I, (3b,21b)-12-oleanene-3,21,28-triol 28-[arabinosyl-(1→3)-arabinosyl-(1→3)-arabinoside], Ginsenoside Rg3, Vinaginsenoside R1, Momordin Ia, Ginsenoside Rg5, and Ginsenoside Rh2.

### 2.2. Network Analysis of the Increased Compounds

#### 2.2.1. Target Prediction

**Figure 5:**
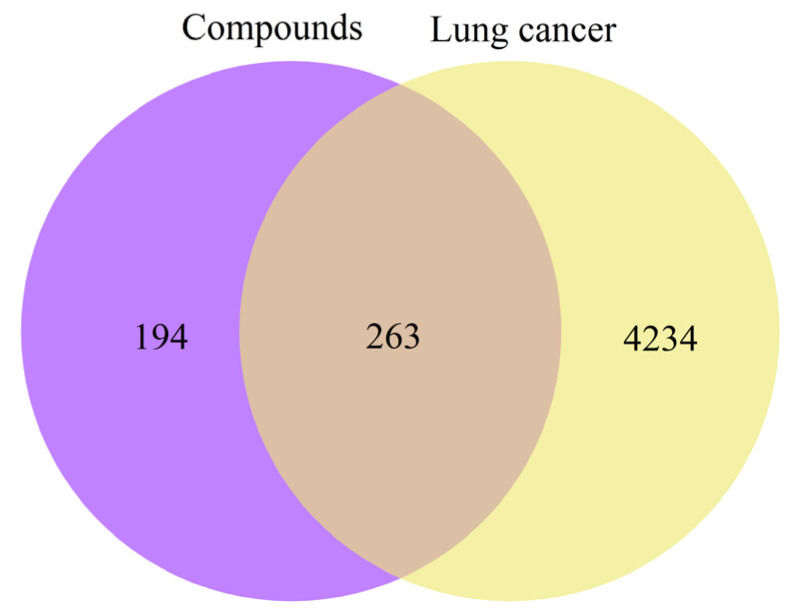
Venn diagram of potential targets.

PharmMapper and SwissTargetPrediction collectively identified 457 potential targets associated with the upregulated compounds, while GeneCards, TTD, and OMIM yielded 4497 lung cancer–related targets. The intersection of these two datasets resulted in 263 common targets, as depicted in the Venn diagram (Figure 5).

#### 2.2.2. Protein–Protein Interaction (PPI) Network Construction

**Figure 6:**
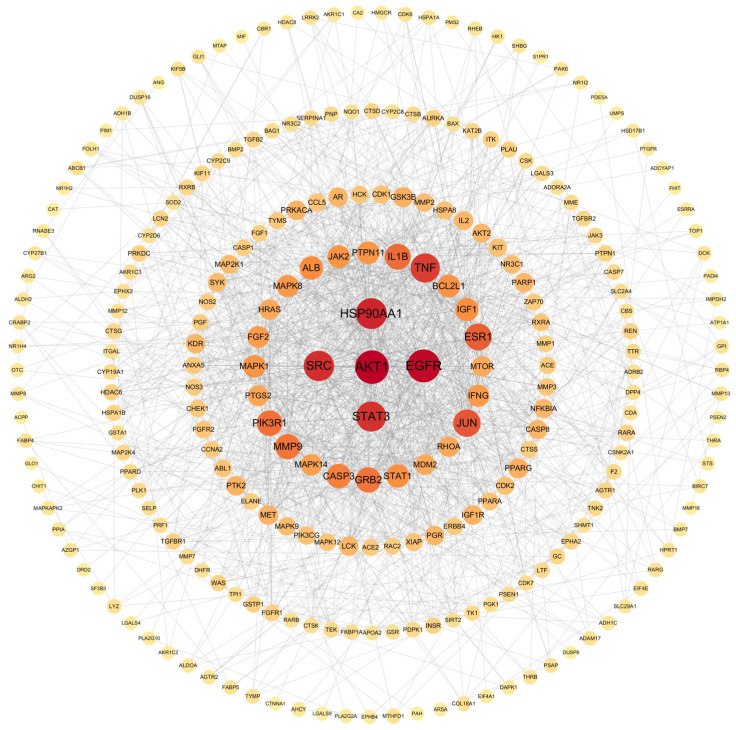
PPI network diagram.

A PPI network was constructed by importing the 263 shared targets into the STRING 11.5 platform. Topological analysis was subsequently performed in Cytoscape 3.9.1 using degree values as the screening parameter. As shown in Figure 6, AKT1, EGFR, HSP90AA1, SRC, and STAT3 exhibited the highest degree values and were identified as core therapeutic targets for lung cancer.

#### 2.2.3. Functional Enrichment Analysis of Gene Ontology (GO) and Kyoto Encyclopedia of Genes and Genomes (KEGG) Pathways

**Figure 7:**
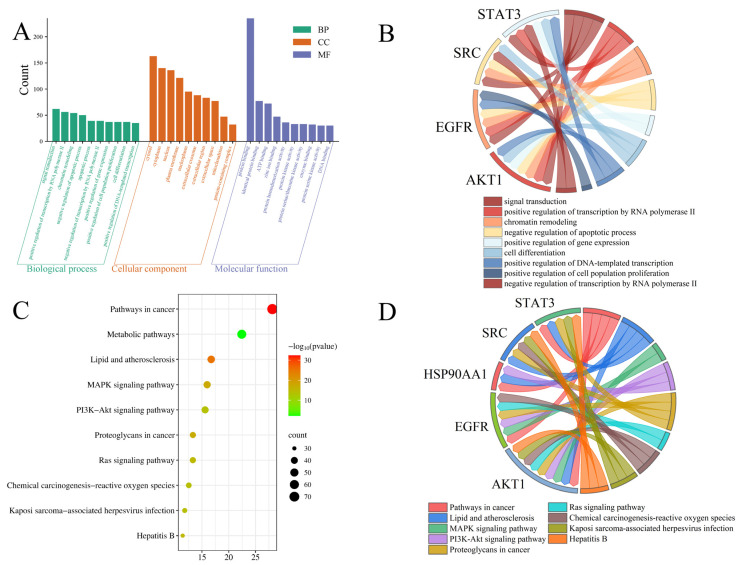
Enrichment analysis of targets. (**A**) GO function enrichment analysis. (**B**) The relationship between the targets and the BPs. (**C**) KEGG pathway enrichment. (**D**) The relationship between the targets and the pathways.

GO and KEGG enrichment analyses were performed using the DAVID database. GO analysis identified 1213 significantly enriched terms, and the top 10 terms from each GO category are shown in Figure 7A. For biological processes (BP), the top enriched terms included signal transduction, positive regulation of transcription by RNA polymerase II, chromatin remodeling, negative regulation of apoptotic process, apoptotic process, negative regulation of transcription by RNA polymerase II, positive regulation of gene expression, positive regulation of cell population proliferation, cell differentiation, and positive regulation of DNA-templated transcription. The enriched cellular component (CC) terms comprised cytosol, cytoplasm, nucleus, plasma membrane, nucleoplasm, extracellular exosome, extracellular region, extracellular space, mitochondrion, and protein-containing complex. The top molecular function (MF) terms included protein binding, identical protein binding, ATP binding, zinc ion binding, protein homodimerization activity, protein kinase activity, protein serine/threonine kinase activity, enzyme binding, protein serine kinase activity, and DNA binding. Among the top enriched BP terms, the identified core targets demonstrated broad functional involvement (Figure 7B). For example, AKT1 participated in seven biological processes, EGFR and STAT3 were involved in six, and SRC contributed to five biological processes, highlighting their essential roles in mediating the anti-tumor effects of steamed PJR.

KEGG pathway enrichment analysis identified 179 significantly enriched pathways, with the top 10 visualized in Figure 7C. These included pathways in cancer, metabolic pathways, lipid and atherosclerosis, MAPK signaling pathway, PI3K-Akt signaling pathway, proteoglycans in cancer, Ras signaling pathway, chemical carcinogenesis-reactive oxygen species, Kaposi sarcoma-associated herpesvirus infection, and hepatitis B. Among these, the MAPK, PI3K-Akt, and Ras signaling pathways appeared to be the major pathways associated with the anti-lung cancer effects of the identified metabolites. The associations between core targets and enriched pathways are illustrated in Figure 7D. Multiple core targets were involved in key signaling pathways. For instance, AKT1 and EGFR were implicated in both the MAPK and Ras signaling pathways, while AKT1, EGFR, and HSP90AA1 were involved in the PI3K-Akt signaling pathway.

#### 2.2.4. The “Component-Target-Pathway” Network

**Figure 8:**
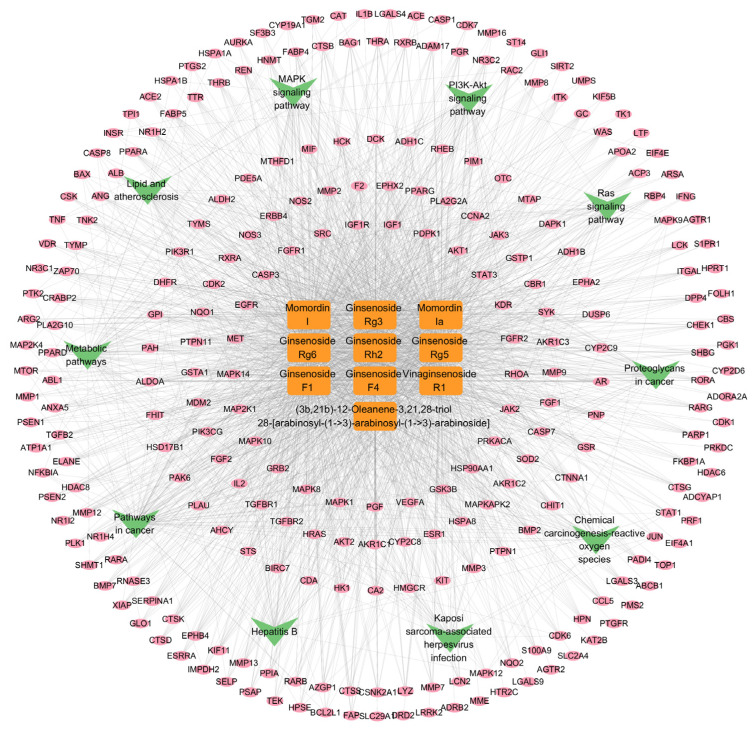
The “component-target-pathway” network.

The “component-target-pathway” network was constructed using Cytoscape 3.9.1, and the resulting visualization is depicted in Figure 8. In the network, orange rectangular nodes represent differential metabolite components, pink elliptical nodes denote predicted targets, and green V-shaped nodes indicate enriched signaling pathways. This network clearly demonstrates that the differential metabolites may exert anti-lung cancer effects through multi-component, multi-target interactions and via the modulation of multiple signaling pathways.

### 2.3. Molecular Docking Analysis

The reliability of the molecular docking procedure was confirmed through re-docking experiments. The root mean square deviation (RMSD) values between the re-docked ligands and the corresponding co-crystallized ligands were 0.622 Å for AKT1, 0.694 Å for EGFR, 1.007 Å for HSP90AA1, 0.578 Å for SRC, and 0.920 Å for STAT3. All RMSD values were well below the threshold of 2.0 Å, indicating that the docking protocol was sufficiently accurate for predicting ligand–protein binding modes.

**Figure 9:**
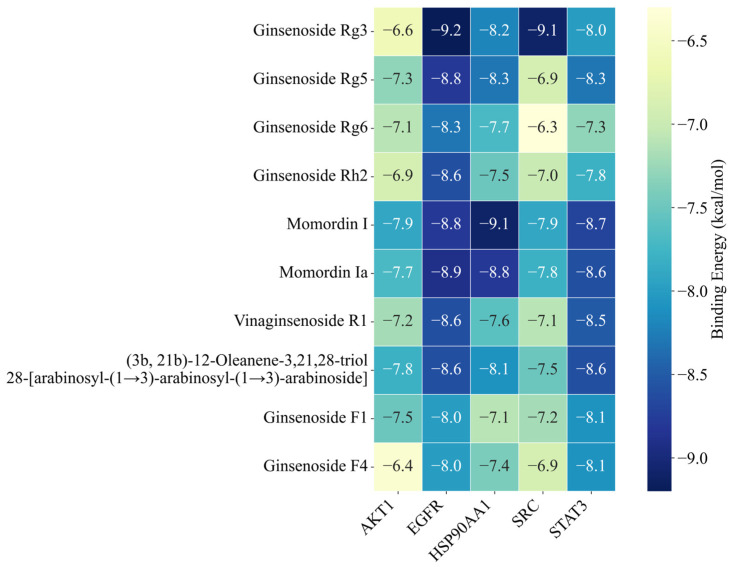
Heatmap of binding affinity values. The numbers indicate the binding energy in kcal/mol. Darker blue indicates stronger binding affinity.

**Figure 10:**
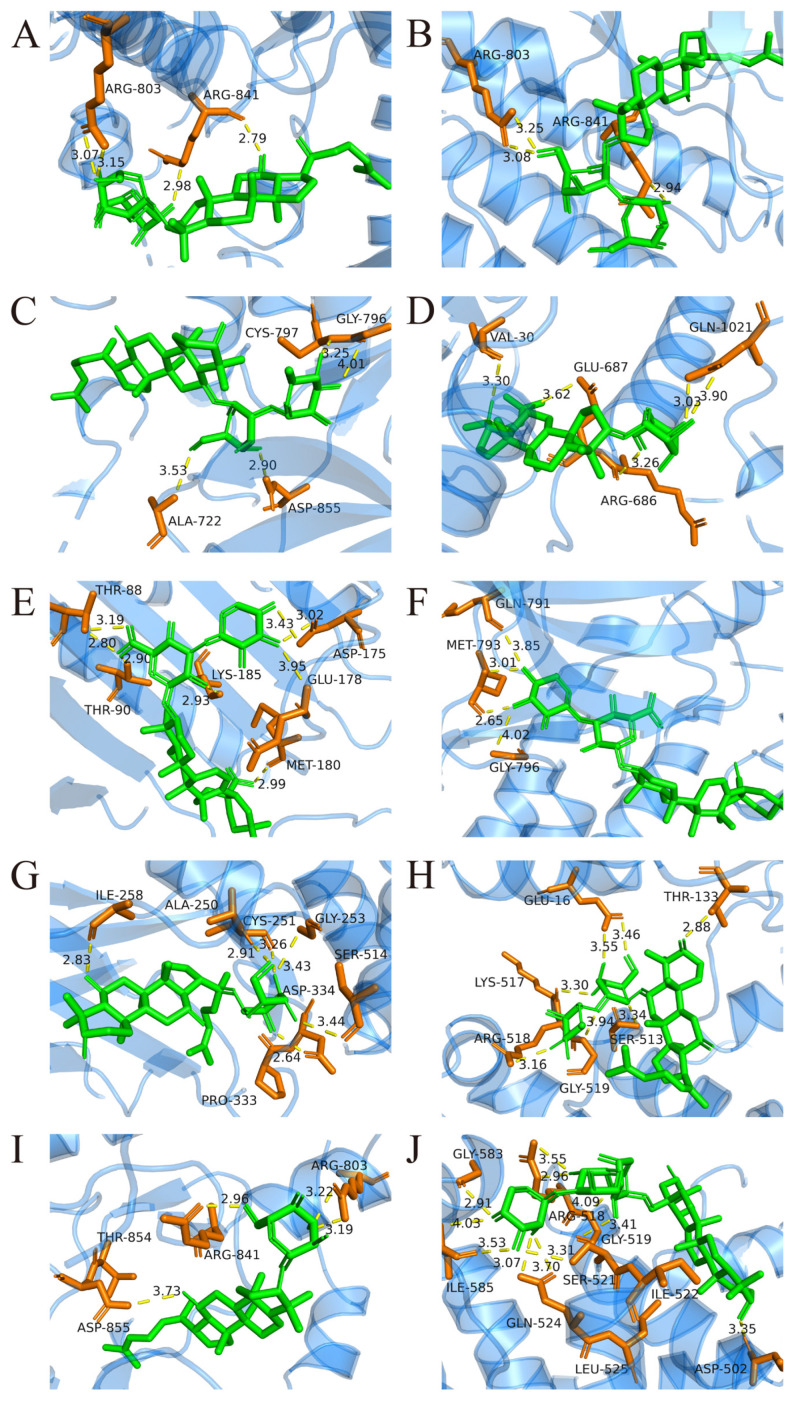
Molecular docking between key components and core targets. Yellow dashed lines represent hydrogen bond interactions, and the adjacent numbers indicate bond lengths in Ångströms (Å). (**A**) Ginsenoside Rg3-EGFR. (**B**) Ginsenoside Rg5-EGFR. (**C**) Ginsenoside Rg6-EGFR. (**D**) Ginsenoside Rh2-EGFR. (**E**) Momordin I-HSP90AA1. (**F**) Momordin Ia-EGFR. (**G**) Ginsenoside F1-STAT3. (**H**) Ginsenoside F4-STAT3. (**I**) Vinaginsenoside R1-EGFR. (**J**) (3b,21b)-12-Oleanene-3,21,28-triol 28-[arabinosyl-(1→3)-arabinosyl-(1→3)-arabinoside]-STAT3.

The upregulated metabolites in steamed PJR, including Ginsenosides Rg3, Rg5, Rg6, Rh2, F1, F4, Vinaginsenoside R1, Momordin I and Ia, and (3b,21b)-12-oleanene-3,21,28-triol 28-[arabinosyl-(1→3)-arabinosyl-(1→3)-arabinoside], were docked with the core targets using AutoDock Vina 1.5.6. Binding affinities were evaluated based on docking scores, with more negative values indicating stronger predicted interactions. As shown in Figure 9, all compounds exhibited binding energies lower than −5 kcal/mol with their respective targets, suggesting strong affinity and favorable binding. Docking conformations were visualized using PyMOL 2.4.0, and representative interaction diagrams are shown in Figure 10. Numerous binding sites were observed between the compounds and core target proteins. For example, Ginsenoside Rg3 interacted with EGFR via residues ARG-841 and ARG-803. Collectively, the docking results indicate that the upregulated metabolites generated during PJR steaming can bind with high affinity to key anti-lung cancer targets, supporting their potential therapeutic relevance.

### 2.4. MD Simulation Analysis

**Figure 11:**
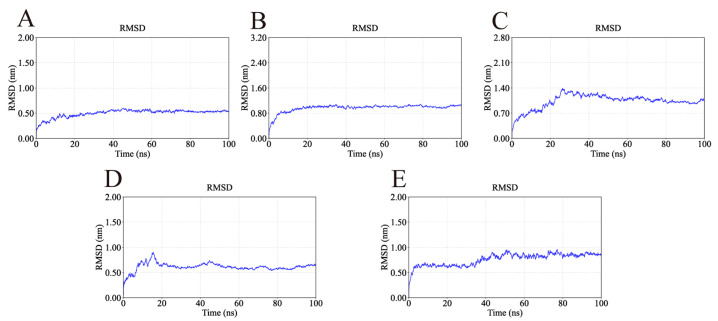
RMSD values of the complexes during molecular dynamics simulations. The flattening of the curves indicates that all protein-ligand complexes reached a dynamic equilibrium state and maintained structural stability throughout the simulation. (**A**) RMSD of AKT1-Ginsenoside F1. (**B**) RMSD of EGFR-Ginsenoside Rh2. (**C**) RMSD of HSP90AA1-Ginsenoside Rg6. (**D**) RMSD of SRC-Ginsenoside Rg3. (**E**) RMSD of STAT3-Ginsenoside F4.

The RMSD is a key indicator for assessing the conformational stability of protein-ligand complexes, with smaller deviations reflecting higher stability. The RMSD profiles of the simulated complexes are presented in Figure 11. As shown in Figure 11, the AKT1-Ginsenoside F1, EGFR-Ginsenoside Rh2, HSP90AA1-Ginsenoside Rg6, SRC-Ginsenoside Rg3, and STAT3-Ginsenoside F4 complexes reached equilibrium at approximately 20 ns, 20 ns, 40 ns, 20 ns, and 40 ns, respectively. Their RMSD values subsequently stabilized around 0.50 nm, 0.95 nm, 1.10 nm, 0.60 nm, and 0.90 nm, suggesting that all five complexes maintained stable conformations during the simulation. Although the HSP90AA1-Ginsenoside Rg6 complex exhibited a relatively high RMSD (≈1.1 nm), visual inspection of the trajectory revealed this was primarily due to the significant flexibility of loop regions and the N/C-terminals. Critically, the core binding domain remained structurally stable throughout the simulation, preserving key interactions with the ligand.

**Figure 12:**
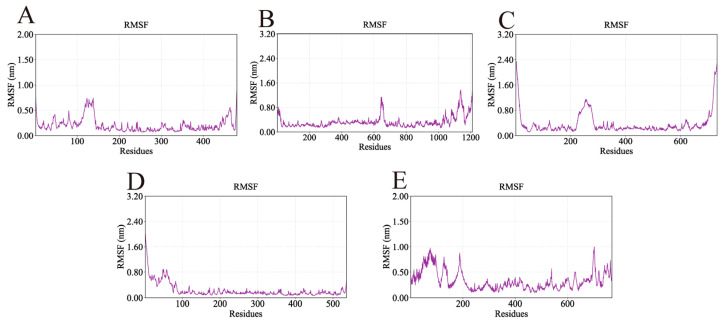
RMSF values of the complexes during molecular dynamics simulations. Lower RMSF values indicate regions of structural rigidity where the ligand binds, while higher peaks correspond to flexible loop regions of the proteins. (**A**) RMSF of AKT1-Ginsenoside F1, (**B**) RMSF of EGFR-Ginsenoside Rh2, (**C**) RMSF of HSP90AA1-Ginsenoside Rg6, (**D**) RMSF of SRC-Ginsenoside Rg3, (**E**) RMSF of STAT3-Ginsenoside F4.

The root mean square fluctuation (RMSF) was used to assess residue-level flexibility, with higher values indicating more pronounced atomic fluctuations. The RMSF profiles of the five complexes are shown in Figure 12. Overall, RMSF values were mostly below 1 nm, demonstrating that the ligands exerted minimal influence on the conformational flexibility and global stability of the proteins. Importantly, the key binding residues identified from docking exhibited low fluctuations, further supporting the stability of the ligand–protein interactions. For example, the AKT1-Ginsenoside F1 complex showed low RMSF values for TYR-175 (0.18 nm), TYR-176 (0.10 nm), LYS-241 (0.26 nm), and GLU-432 (0.20 nm). Similarly low fluctuations were observed for the key residues of EGFR-Ginsenoside Rh2 (VAL-30, ARG-686, GLU-687, GLN-1021), HSP90AA1-Ginsenoside Rg6 (GLU-547, GLU-550, GLN-561, ASN-590), SRC-Ginsenoside Rg3 (TYR-93, PHE-153, LYS-252, GLN-254), and STAT3-Ginsenoside F4 (GLU-16, THR-133, SER-513, LYS-517, ARG-518, GLY-519).

**Figure 13:**
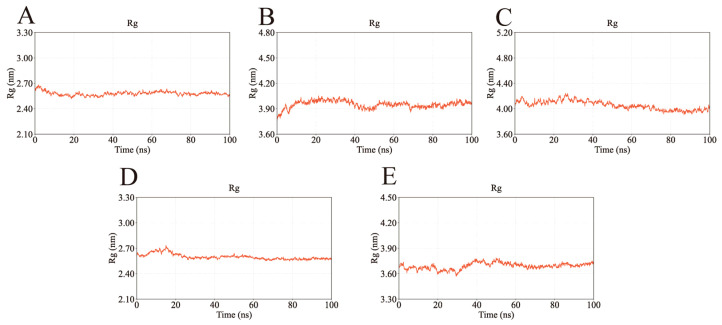
Rg values of the complexes during molecular dynamics simulations. The stable Rg values suggest that the complexes remained compact and did not undergo significant unfolding during the binding process. (**A**) Rg of AKT1-Ginsenoside F1. (**B**) Rg of EGFR-Ginsenoside Rh2. (**C**) Rg of HSP90AA1-Ginsenoside Rg6. (**D**) Rg of SRC-Ginsenoside Rg3. (**E**) Rg of STAT3-Ginsenoside F4.

The radius of gyration (Rg), which reflects protein compactness, was used to evaluate overall structural stability. As shown in Figure 13, the Rg values of all complexes remained stable throughout the simulation, converging at approximately 2.60 nm (AKT1), 4.00 nm (EGFR), 4.05 nm (HSP90AA1), 2.60 nm (SRC), and 3.70 nm (STAT3). These results indicate consistent structural compactness and support the stable binding of the ligands.

**Figure 14:**
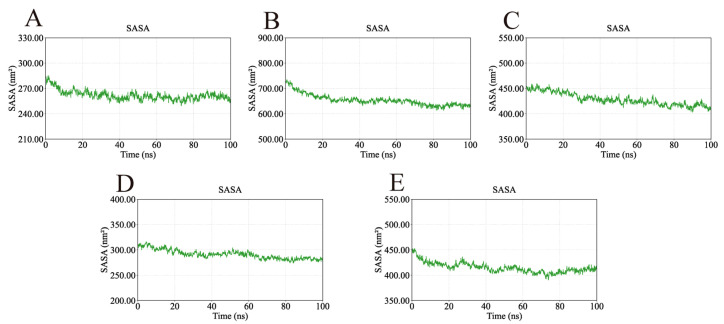
SASA values of the complexes during molecular dynamics simulations. The consistency of the SASA values further confirms the structural stability of the complexes. (**A**) SASA of AKT1-Ginsenoside F1. (**B**) SASA of EGFR-Ginsenoside Rh2. (**C**) SASA of HSP90AA1-Ginsenoside Rg6. (**D**) SASA of SRC-Ginsenoside Rg3. (**E**) SASA of STAT3-Ginsenoside F4.

The solvent-accessible surface area (SASA) was monitored to assess protein surface exposure (Figure 14). The SASA values of the five complexes exhibited only minor fluctuations—249.52–285.01 nm^2^ (AKT1), 612.98–735.21 nm^2^ (EGFR), 403.02–459.35 nm^2^ (HSP90AA1), 273.91–316.06 nm^2^ (SRC), and 390.56–452.91 nm^2^ (STAT3)—further confirming their structural stability during the simulation period.

**Figure 15:**
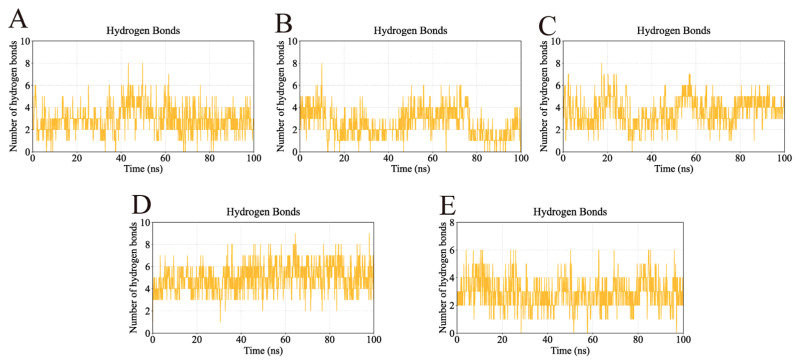
Number of hydrogen bonds in the complexes during molecular dynamics simulations. The continuous presence of hydrogen bonds indicates strong and persistent interactions between the ligands and their target proteins. (**A**) Number of hydrogen bonds in AKT1-Ginsenoside F1. (**B**) Number of hydrogen bonds in EGFR-Ginsenoside Rh2. (**C**) Number of hydrogen bonds in HSP90AA1-Ginsenoside Rg6. (**D**) Number of hydrogen bonds in SRC-Ginsenoside Rg3. (**E**) Number of hydrogen bonds in STAT3-Ginsenoside F4.

Hydrogen bonding interactions, which play a critical role in stabilizing ligand–protein complexes, were also analyzed (Figure 15). The AKT1-Ginsenoside F1, EGFR-Ginsenoside Rh2, HSP90AA1-Ginsenoside Rg6, SRC-Ginsenoside Rg3, and STAT3-Ginsenoside F4 complexes formed up to 8, 8, 8, 9, and 6 hydrogen bonds, respectively. Their average hydrogen bond counts were 4, 3, 4, 6, and 4, indicating strong and consistent interactions between each ligand and its target protein.

**Figure 16:**
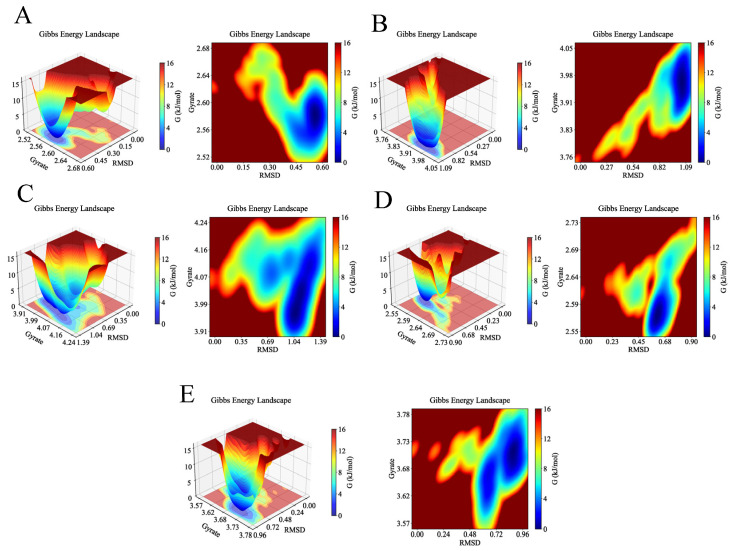
Gibbs free energy of the complexes during molecular dynamics simulations. The deep blue basins represent the lowest energy conformations, indicating the most stable binding states for each complex. (**A**) 3D and 2D Gibbs free energy landscapes of AKT1-Ginsenoside F1. (**B**) 3D and 2D Gibbs free energy landscapes of EGFR-Ginsenoside Rh2. (**C**) 3D and 2D Gibbs free energy landscapes of HSP90AA1-Ginsenoside Rg6. (**D**) 3D and 2D Gibbs free energy landscapes of SRC-Ginsenoside Rg3. (**E**) 3D and 2D Gibbs free energy landscapes of STAT3-Ginsenoside F4.

Finally, Gibbs free energy landscapes were computed based on RMSD and Rg values to assess conformational stability. As shown in Figure 16, the AKT1-Ginsenoside F1, EGFR-Ginsenoside Rh2, HSP90AA1-Ginsenoside Rg6, SRC-Ginsenoside Rg3, and STAT3-Ginsenoside F4 complexes exhibited lower free energy states within the Rg ranges of 2.56–2.60 nm, 3.92–4.00 nm, 3.93–4.05 nm, 2.55–2.60 nm, and 3.68–3.73 nm, respectively, and RMSD ranges of 0.50–0.60 nm, 0.96–1.09 nm, 0.91–1.12 nm, 0.55–0.65 nm, and 0.84–0.96 nm. These results reveal that the small molecules and targets can stably bind.

### 2.5. Gene Expression Omnibus (GEO) Database Validation

**Figure 17:**
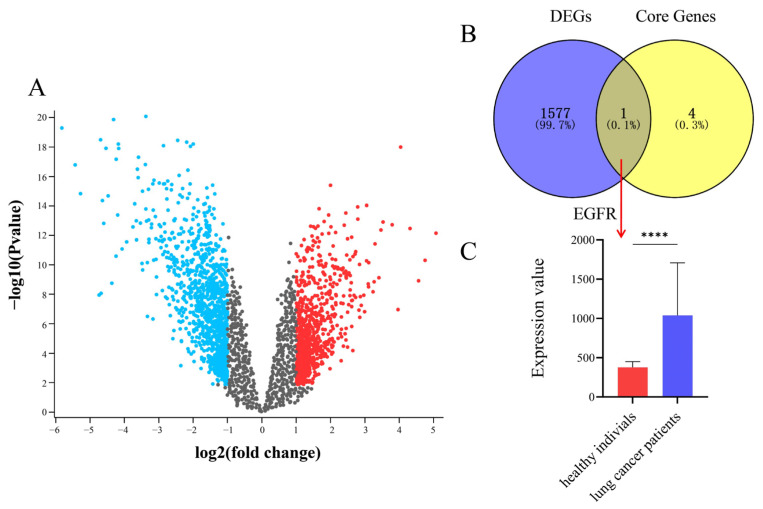
Validation of core targets from datasets of GEO. (**A**) Volcano map. The red and blue dots indicate significantly upregulated and downregulated genes, respectively, while grey dots represent genes with no significant changes. (**B**) Venn diagram of DEGs and core genes. (**C**) EGFR expression levels between healthy individuals and lung cancer patients, **** *p* < 0.0001.

Differentially expressed genes (DEGs) in the GSE7670 dataset were analyzed using the GEO2R tool (GEO database) with thresholds of *p* < 0.05 and |logFC| > 1. A volcano plot was generated to visualize the distribution of DEGs, where red and blue dots indicated upregulated and downregulated genes, respectively (Figure 17A). A Venn diagram illustrating the overlap between DEGs and core targets is shown in Figure 17B. As demonstrated, EGFR was identified as the intersecting core target. Further validation of EGFR expression revealed significantly elevated EGFR levels in lung cancer patients compared with healthy individuals (Figure 17C).

### 2.6. Cellular Viability Under Drug-Containing Serum Treatment

**Figure 18:**
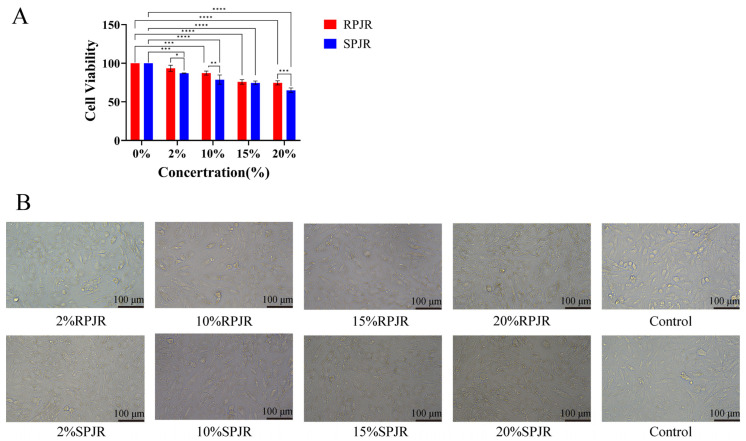
The Effects of Medicinal Sera from RPJR and SPJR on A549 Cells. (**A**) Effect of drug-containing sera on cell activity. Statistical significance was determined using one-way ANOVA. Data are presented as mean ± SD (*n* = 3). * *p* < 0.05, ** *p* < 0.01, *** *p* < 0.001, **** *p* < 0.0001. (**B**) Effect of drug-containing sera on morphological changes in cells. Scale bar: 100 μm.

CCK-8 assays were performed to evaluate A549 cell viability following treatment with serial dilutions of PJR-containing serum (Figure 18A). Relative to the control group, increasing concentrations of PJR-containing serum resulted in a significant reduction in cell viability (*p* < 0.001), except for the 2% raw PJR serum group. These findings indicate that both raw and steamed PJR markedly inhibited A549 cell proliferation. Moreover, at 10% and 20% serum concentrations, steamed PJR exerted significantly stronger inhibitory effects than raw PJR (*p* < 0.05), demonstrating that steaming enhances the anti-lung cancer activity of PJR.

### 2.7. Cellular Morphology Alterations Induced by Drug-Containing Serum

After 24 h of treatment with raw or steamed PJR drug-containing sera at various concentrations, notable morphological changes were observed in A549 cells (Figure 18B). As seen in Figure 18B, control cells displayed a normal adherent morphology with well-defined cellular contours, whereas cells exposed to PJR-containing serum exhibited dose-dependent shrinkage and reduced intercellular boundaries. Maximal morphological disruption with pronounced cell wrinkling occurred at 20% steamed PJR serum, manifesting stronger inhibitory effects of steamed PJR on A549 cells.

## 3. Discussion

Current therapeutic strategies for lung cancer include surgical resection, radiotherapy, chemotherapy, and targeted drug therapy. Although these approaches contribute to disease management, substantial limitations remain regarding long-term efficacy. For instance, 30–50% of patients experience recurrence following surgical resection [32]. Conventional chemotherapy provides limited benefit, with the 5-year survival rate remaining below 15% [33]. Prolonged exposure to chemotherapeutic agents may also induce tumor cell adaptation, activating pro-survival signaling pathways and promoting multidrug resistance, ultimately leading to therapeutic failure [34]. Radiotherapy, while effective in eliminating tumor cells, is associated with significant adverse effects such as radiation-induced lung injury (RILI) [35] and radiation-induced heart damage (RIHD) [36]. Targeted therapies directed at specific molecular alterations, such as epidermal growth factor receptor (EGFR) mutations, have markedly improved patient survival. However, not all patients harbor targetable oncogenic drivers, and acquired resistance to targeted agents represents a major clinical obstacle [37]. In recent years, TCM has shown promising efficacy in cancer treatment through mechanisms including immune modulation and anti-inflammatory effects, which collectively enhance host immunity and suppress tumor proliferation. Increasing evidence indicates that tumor progression is closely associated with malignant cell proliferation, metastasis, and invasion; thus, targeting these processes may effectively inhibit tumor growth and dissemination, thereby improving patient outcomes [38,39,40]. PJR is a valuable TCM herb recognized for its notable anti-tumor potential. Its anti-cancer activity is primarily attributed to its rich saponin content, which exhibits inhibitory effects against various cancer cell types, including prostate, liver, gastric, lung, and ovarian cancers. These effects are mediated through mechanisms such as induction of apoptosis, suppression of cell migration and invasion, and modulation of oncogene expression [41].

Metabolomics was employed in this study and led to the identification of 22 differential saponin compounds between raw and steamed PJR. Among these metabolites, 12 decreased in abundance, whereas 10 increased significantly after steaming. Under high-temperature steaming conditions, polar saponins such as ginsenosides Rd and Re undergo structural transformations, including dehydration and deglycosylation, resulting in their conversion into less polar rare saponins such as Rk1, Rg3, Rh2, and Rg5. Ten metabolites were markedly upregulated after processing, and notably, many of these rare saponins (e.g., ginsenosides Rg3, Rh2, and Rg5) are well-documented for their potent anti-tumor pharmacological activities, exhibiting inhibitory effects against various cancer cell types [42,43].

These significantly upregulated metabolites in steamed PJR likely constitute the material basis underlying its enhanced pharmacological activity compared with raw PJR. Consequently, network pharmacology analysis was conducted on these key differential components. Five core protein targets, AKT1, EGFR, HSP90AA1, SRC, and STAT3, were identified as potential mediators of steamed PJR’s anti-lung cancer effects. SRC plays a pivotal role in regulating cell proliferation, migration, and survival. Aberrant SRC activation, frequently observed in multiple cancers, induces phosphorylation at the Y419 residue, thereby enhancing its kinase activity and promoting tumor cell proliferation, invasion, and metastasis [44]. EGFR, a receptor tyrosine kinase (RTK), forms homo- or heterodimers with other RTKs such as HER2, leading to autophosphorylation and activation of downstream signaling cascades [45]. These cascades, RAS-RAF-MEK-MAPK, PI3K-PTEN-AKT, and STAT pathways, collectively drive oncogenic processes including uncontrolled proliferation, angiogenesis, metastasis, and inhibition of apoptosis [46,47]. In addition, EGFR serves as a regulatory factor for SRC, modulating SRC/STAT3 and SRC/ERK pathways [48] and participating in angiogenesis [49]. AKT1, one of the three highly homologous isoforms of the AKT serine/threonine kinase family, governs essential cellular functions such as metabolism, proliferation, survival, and angiogenesis [50]. Upon activation by LEP, STAT3 regulates apoptosis by transcriptionally upregulating BIRC5 expression [51]. HSP90AA1 modulates cell cycle-related proteins and influences cell cycle progression and proliferation. In cancer cells, HSP90AA1 is often overactivated, contributing to tumor growth and survival [52]. Collectively, these findings suggest that AKT1, EGFR, HSP90AA1, SRC, and STAT3 represent important therapeutic targets for anti-lung cancer intervention. Therefore, exploring the interactions between the upregulated metabolites and these core proteins is essential for elucidating their pharmacological potential.

Further molecular docking analysis demonstrated strong binding capabilities between the 10 differential metabolites from steamed PJR and the core protein targets, with all binding affinities below −5 kcal/mol. These results indicate that the upregulated metabolites may serve as key anti-lung cancer constituents and support the reliability of the network pharmacology predictions. MD simulations further validated these findings, showing that the key complexes (AKT1-Ginsenoside F1, EGFR-Ginsenoside Rh2, HSP90AA1-Ginsenoside Rg6, SRC-Ginsenoside Rg3, and STAT3-Ginsenoside F4) maintained stable binding conformations. Both RMSD and RMSF values remained low throughout the simulations, and Gibbs free energy analyses confirmed their thermodynamic stability. Collectively, these results strongly corroborate the predictions derived from molecular docking. Additionally, EGFR was found to be significantly overexpressed in lung cancer patients, as validated by the GEO dataset GSE7670, further supporting its role as a core therapeutic target.

GO enrichment analysis revealed substantial enrichment of therapeutic targets across BP, CC, and MF, suggesting that the anti-lung cancer effects of steamed PJR may involve the modulation of multiple cellular pathways and functional processes. KEGG pathway analysis showed that the differential metabolites associated with lung cancer treatment were primarily linked to pathways involved in immune responses, cell proliferation, differentiation, stress response, and energy metabolism. Among the top 10 enriched pathways, the PI3K-Akt signaling pathway was prominently represented, underscoring its central role in mediating the therapeutic effects of steamed PJR. The PI3K-Akt pathway is a critical intracellular signaling cascade that regulates diverse physiological and pathological processes, including cell proliferation, survival, differentiation, growth, and apoptosis [53]. This pathway is known to exert significant influence within the tumor microenvironment by promoting angiogenesis and recruiting inflammatory factors [54]. Mechanistically, PI3K is activated upon growth factor stimulation, catalyzing the conversion of PIP2 to PIP3. PIP3 subsequently activates Akt, which phosphorylates downstream substrates to promote cellular growth, proliferation, and survival [55].

In A549 lung cancer cells treated with drug-containing sera, CCK-8 assays showed that both raw and steamed PJR reduced cell viability in a dose-dependent manner. As the primary bioactive constituents of PJR, saponins undergo structural transformations during high-temperature steaming, converting into rare saponins with stronger pharmacological activities. This transformation likely contributes to the enhanced anti-tumor efficacy of steamed PJR. Notably, sera containing 10% and 20% steamed PJR demonstrated significantly greater inhibitory effects than equivalent concentrations of raw PJR serum, suggesting that the elevated rare saponin content in steamed PJR underlies its superior anti-lung cancer activity. These findings indicate that steamed PJR holds strong potential as a therapeutic candidate for lung cancer and merits further investigation.

Previous studies have shown that thermal processing of *Panax* species, such as *Panax ginseng* and *Panax notoginseng*, facilitates the conversion of major saponins into rare saponins [23,56], which exhibit potent anti-lung cancer activities [57]. Although this suggests a possible genus-wide trend, PJR is a distinct species with a unique phytochemical profile that differs markedly from that of *P. ginseng* and *P. notoginseng*. Consequently, results obtained from other *Panax* species cannot be directly extrapolated to PJR due to interspecies differences in saponin composition and abundance. Our study addresses this gap by providing direct experimental evidence specific to PJR. Consistent with established findings in related species, our results demonstrate that steaming significantly enhances the anti-lung cancer effects of PJR, accompanied by a substantial increase in rare saponin content—thus confirming that this thermal transformation pattern also applies to PJR. Moreover, by employing an integrated strategy encompassing metabolomics, network pharmacology, molecular docking, MD simulations, and cellular validation, we successfully delineated the specific material basis and molecular targets underlying the enhanced anti-tumor activity of steamed PJR. These findings clarify the distinct pharmacological value of steamed PJR and further support its potential as a unique medicinal resource.

While this study successfully integrated metabolomics, network pharmacology, and MD simulations to elucidate the differences between raw and steamed PJR, several limitations should be acknowledged to guide future research. Firstly, the biological validation was conducted primarily using the A549 lung cancer cell line. Although A549 is a representative NSCLC model with high expression of core targets such as EGFR, extending validation to additional lung cancer cell lines, such as H1299 or PC-9, would enhance the generalizability and robustness of the findings. Secondly, the current experimental scope focused on assessing antiproliferative effects through morphological observation and CCK-8-based cell viability assays. Consequently, key mechanistic processes, including apoptosis induction, cell cycle arrest, and the inhibition of migration and invasion, were inferred from computational analyses but not experimentally confirmed. These mechanisms should be further validated using standard biochemical assays such as flow cytometry, Western blotting, and migration/invasion assays. Future studies will therefore prioritize verifying the regulation of predicted core signaling pathways, particularly the PI3K-AKT and MAPK axes, and expanding the evaluation of steamed PJR’s anti-lung cancer potential across a broader array of biological models.

## 4. Materials and Methods

### 4.1. Chemicals and Reagents

Notoginsenoside R1 (Lot: wkq22112204), Ginsenoside Rg3 (Lot: wkq23041807), Ginsenoside Rd (Lot: wkq23050807), and Ginsenoside Rh2 (Lot: wkq22092310) were obtained from Sichuan Weikeqi Biotech Technology Co., Ltd. (Chengdu, China). LC-MS-grade acetonitrile was purchased from Merck KGaA (Darmstadt, Germany), LC-MS-grade formic acid was provided by CNW Technologies GmbH (Düsseldorf, Germany), and analytical-grade methanol was supplied by Sinopharm Chemical Reagent Co., Ltd. (Shanghai, China). Two batches of PJR (sourced from Sichuan and Hunan) were authenticated as *Panax japonicus* C.A.Mey. rhizomes by Associate Professor Wang Zhi from the Hunan University of Chinese Medicine.

### 4.2. Metabolomic Profiling of Raw and Steamed PJR

#### 4.2.1. Preparation of Standard Solutions

Accurately weighed quantities of ginsenosides Rg3, Rd, Rh2, and notoginsenoside R1 were dissolved in 70% methanol in separate 10 mL volumetric flasks to prepare individual stock solutions. These stock solutions were subsequently diluted with 70% methanol to yield working standard solutions at the following concentrations: 0.91 μg/mL (Rg3), 0.99 μg/mL (Rd), 0.90 μg/mL (Rh2), and 1.10 μg/mL (notoginsenoside R1).

#### 4.2.2. Preparation of Test Products

The steaming procedure for PJR was conducted as follows: A 20 g portion of PJR was soaked in water for 2 h in a glass container to ensure moisture equilibration, then subjected to high-pressure steaming at 125 °C for 3 h in an autoclave. The steamed material was subsequently dried at 80 °C to a constant weight.

For extraction, 1.0 g of raw or steamed PJR powder (passed through a 40-mesh sieve) was accurately weighed and placed into a 100 mL stoppered conical flask. Each sample was extracted with 50 mL of 70% methanol using a water bath reflux system for 1 h. After cooling to room temperature, the flasks were reweighed, and any methanol loss was compensated by adding additional 70% methanol. The samples were then vortex-mixed and centrifuged at 5000 rpm for 20 min at 4 °C, and the resulting supernatants were collected for LC-MS analysis.

Four biological replicates were prepared for both raw and steamed PJR, yielding a total of 16 samples. Quality control (QC) samples were prepared by pooling equal masses of raw and steamed extracts, with four biological replicates generated. During the analytical run, a QC sample was injected after every five test samples to monitor instrument stability, reproducibility, and precision throughout the sequence.

#### 4.2.3. UPLC-Q-TOF-MS Conditions

UPLC-Q-TOF-MS analysis was performed using a Waters ACQUITY H-Class Ultra Performance LC system coupled to an Xev G2-XS Q-TOF mass spectrometer (Waters, Milford, MA, USA). Data acquisition and processing were conducted using MassLynx 4.2 software.

Chromatographic separation was achieved on a Waters ACQUITY^TM^ Premier HSS T3 VanGuard™ FIT column (2.1 × 100 mm, 1.8 μm). The mobile phase consisted of 0.1% formic acid in water (A) and acetonitrile (B). The gradient elution program was as follows: 0–2 min, 8–8% B; 2–3 min, 8–13% B; 3–5 min, 13–20.5% B; 5–11 min, 20.5–26% B; 11–13 min, 26–27% B; 13–15 min, 27–31% B; 15–25 min, 31–31% B; 25–30 min, 31–37% B; 30–40 min, 37–43% B; 40–45 min, 43–50% B; 45–51 min, 50–59% B; 51–52 min, 59–75% B; 52–60 min, 75–90% B; 60–65 min, 90–98% B. The flow rate was maintained at 0.3 mL/min, the column temperature at 35 °C, and the injection volume was 1 μL.

Mass spectrometry data were acquired in electrospray ionization (ESI) negative-ion mode under the following conditions: cone voltage, 30 V; capillary voltage, 3 kV; ion source temperature, 120 °C; desolvation temperature, 400 °C; desolvation gas flow rate, 50 L/h. Data were collected in MSE mode over a 70 min acquisition period, with a mass range of 100–1500 mDa. Collision energy was set at 40–70 V using high-purity argon as the collision gas. Leucine enkephalin was used for real-time mass calibration.

#### 4.2.4. Data Processing and Analysis

LC-MS raw data obtained from 16 batches of raw and steamed PJR samples were processed using Progenesis QI software (v3.1, Nonlinear Dynamics, Newcastle upon Tyne, UK) for baseline correction, peak detection, peak integration, retention time alignment, and normalization. Subsequent statistical analyses were conducted with SIMCA-P 14.1 software (Umetrics AB, Umeå, Sweden). Unsupervised pattern recognition, including PCA, was employed to distinguish metabolic differences between raw and steamed PJR. OPLS-DA was used to further identify key metabolites responsible for intergroup variation. Model robustness and the absence of overfitting were validated via a permutation test with 200 iterations. Differential metabolites were screened based on variable importance in projection (VIP > 1) from the OPLS-DA model and statistical significance (*p* < 0.05, Student’s *t*-test). Structural identification of metabolites was confirmed through comparison of retention times, elemental compositions, and MS/MS fragmentation patterns with those of authentic reference standards and entries in established metabolomic databases.

### 4.3. Network Pharmacology

#### 4.3.1. Acquisition of Targets for Differential Metabolites

To explore the potential anti-lung cancer targets and mechanisms associated with the significantly upregulated metabolites in steamed PJR, compounds showing marked increases relative to the raw samples were selected for further analysis. Potential target proteins were predicted using the PharmMapper database (http://www.lilab-ecust.cn/pharmmapper/submitfile.html, accessed in 22 January 2025) and the Swiss Target Prediction database (http://www.swisstargetprediction.ch/, accessed in 22 January 2025). In SwissTargetPrediction, only targets with a prediction probability > 0 were retained to ensure broad target coverage. For PharmMapper, the top 300 targets ranked by Fit Score were selected. All predicted targets were standardized to official gene symbols using the UniProt database (https://www.uniprot.org/, accessed in 22 January 2025). Duplicate entries and targets lacking valid standardized gene names were removed to obtain a high-confidence metabolite-target dataset.

#### 4.3.2. Screening of Disease-Associated Targets

Potential therapeutic targets related to lung cancer were retrieved from the GeneCards (https://genecards.org/, accessed in 22 January 2025), TTD (http://db.idrblab.net/ttd/, accessed in 22 January 2025), and OMIM (https://www.omim.org/, accessed in 22 January 2025) databases using “Lung Cancer” as the search term. To ensure high disease relevance, only genes with a GeneCards Relevance Score > 10 were retained. The target lists generated from the three databases were then consolidated to establish a comprehensive set of lung cancer-associated targets.

#### 4.3.3. Construction of a Venn Diagram

The overlap between compound-related targets and disease-associated targets was determined using the VENNY 2.1 online tool (https://bioinfogp.cnb.csic.es/tools/venny/, accessed in 22 January 2025). Subsequently, Venn diagrams were generated using OriginPro 2021 to visually represent the intersecting target sets.

#### 4.3.4. Construction of the PPI Network

Common targets identified from the Venn analysis were imported into the STRING 12.0 database (https://string-db.org/, accessed in 22 January 2025) to construct a PPI network. The resulting network data were further analyzed in Cytoscape 3.9.1, where core targets were extracted based on topological parameters such as degree value.

#### 4.3.5. GO Functional Annotation and KEGG Pathway Enrichment Analysis

Functional enrichment analysis of the shared targets was performed using the DAVID database (https://davidbioinformatics.nih.gov/, accessed in 26 January 2025), covering GO categories (biological process, cellular component, and molecular function) as well as KEGG signaling pathways. Enrichment results were visualized as bubble plots using the Microbiome Analysis Platform (http://www.bioinformatics.com.cn/, accessed in 26 January 2025).

#### 4.3.6. Construction of the Compound-Target-Pathway Network

KEGG-enriched pathways, together with their associated targets and differential metabolites, were imported into Cytoscape 3.9.1 to construct a compound-target-pathway network integrating metabolite changes between raw and steamed PJR, predicted tumor-related targets, and key signaling pathways.

### 4.4. Molecular Docking

Protein structures were preprocessed by removing water molecules and co-crystallized ligands, followed by the addition of polar hydrogens and Kollman charges. Ligand structures were prepared using AutoDock Tools, including hydrogen atom addition and protonation. To verify the reliability of the docking protocol, a re-docking experiment was conducted in which the original co-crystallized ligands were extracted and re-docked into their respective active sites under identical docking parameters. The RMSD between the re-docked ligand pose and the native conformation was then calculated; a docking protocol producing an RMSD < 2.0 Å was considered accurate and reliable.

Molecular docking of the selected metabolites with target proteins was performed using AutoDock Vina, with binding affinity evaluated based on the calculated binding energy. The lowest-energy conformation for each compound was selected for further analysis. Binding interaction diagrams were generated using PyMOL 2.4.0 and the PLIP web server (https://plip-tool.biotec.tu-dresden.de/plip-web/plip/index, accessed in 29 January 2025).

### 4.5. MD Simulation

Metabolites that increased significantly after steaming and ranked among the top five in VIP values were selected for MD simulation. Based on their favorable binding energies and distinct protein targets, the following complexes were chosen for MD analysis: AKT1-Ginsenoside F1, EGFR-Ginsenoside Rh2, HSP90AA1-Ginsenoside Rg6, SRC-Ginsenoside Rg3, and STAT3-Ginsenoside F4.

MD simulations were performed using GROMACS 2023. The protein topology was generated using the Amber99SB-ILDN force field. For the ligands (saponins), topology files were prepared using the General Amber Force Field (GAFF), with partial charges assigned via the AM1-BCC method. Each complex was solvated in a cubic TIP3P water box (10 × 10 × 10 nm^3^), ensuring a minimum distance of 1.2 nm between the protein surface and the box boundary. Counterions (Na^+^/Cl^−^) were added to neutralize the system. Electrostatic interactions were handled using the Particle-Mesh Ewald (PME) method with a 1.0 nm cutoff, and van der Waals interactions were truncated at 1.0 nm. Energy minimization was carried out using the steepest descent algorithm (maximum 50,000 steps). Subsequently, the system underwent equilibration under NVT and NPT ensembles for 100 ps each. Temperature was maintained at 300 K using the V-rescale thermostat, and pressure was controlled at 1 bar using the Berendsen barostat. A 100 ns production run was then performed for each complex. Visualization and analysis of MD trajectories were conducted using Python 3.12.

### 4.6. GEO Database Validation

DEGs between normal and lung cancer tissues in the GSE7670 dataset were identified using the GEO2R online analysis tool available through the GEO database (https://www.ncbi.nlm.nih.gov/geo/, accessed in 17 March 2025). Statistical thresholds were set at *p* < 0.05 and |logFC| > 1. Venn diagram analysis was subsequently performed to determine the overlapping genes between PJR’s core therapeutic targets and the DEGs identified from GSE7670. The expression levels of these shared targets were visualized using corresponding expression plots.

### 4.7. Experimental Verification

#### 4.7.1. Preparation of Experimental Liquid Medicine

A total of 400 g each of raw and steamed PJR powders (passed through a 20-mesh sieve) were soaked for 30 min, followed by two rounds of extraction with 70% ethanol at solvent-to-material ratios of 10-fold and 8-fold, respectively. Reflux extraction was conducted for 3 h during the first extraction and 2 h during the second. The extracts were filtered, combined, and concentrated to remove ethanol. The concentrated solutions were transferred to evaporating dishes and dried to a thick paste using a water bath, after which they were lyophilized to obtain dry powdered extracts. The resulting dry powder was dissolved in a 0.3% carboxymethyl cellulose sodium (CMC-Na) solution to prepare a gavage administration suspension at a final concentration of 0.324 g/mL, calculated based on clinical daily dosage and body surface area conversion guidelines.

#### 4.7.2. Animal Grouping and Drug Administration

Male Sprague-Dawley rats (160 ± 20 g; Hunan Slack Jingda Co., Ltd. (Changsha, China), license ZS-202312120016) were housed under controlled environmental conditions (22–25 °C, 50–70% relative humidity) with a 12 h light-dark cycle. After a 3-day acclimatization period, the animals were randomly assigned to three groups (*n* = 8 per group): blank control, raw PJR treatment, and steamed PJR treatment. Beginning on day 4, rats in the treatment groups received daily intragastric administration of raw or steamed PJR extract (0.324 g/mL), while the blank control group received an equivalent volume of 0.3% CMC-Na solution. All treatments were administered once daily for a total of 7 days.

#### 4.7.3. Preparation of Drug-Containing Serum

One hour after the final administration, blood samples were collected from rats in both the blank and treatment groups. Serum was separated, heat-inactivated at 56 °C for 30 min and subsequently filtered through a 0.22 µm sterile membrane to obtain blank serum and drug-containing serum for in vitro experiments.

#### 4.7.4. Cell Culture and Passage

The A549 human non-small cell lung cancer cell line was obtained from the National Institutes for Food and Drug Control (NIFDC, Beijing, China). Cells were cultured in complete medium consisting of 89% (*v*/*v*) Ham’s F-12K basal medium (Gibco, Waltham, MA, USA), 10% fetal bovine serum (Gibco, USA), and 1% penicillin-streptomycin solution (Solarbio, Beijing, China). Cultures were maintained at 37 °C in a humidified incubator with 5% CO_2_. When cell confluence reached approximately 80%, cells were digested with 0.25% trypsin-EDTA solution (Gibco, USA) and subcultured.

#### 4.7.5. Cell Proliferation Assay

Ten experimental groups were established, including control groups (10% normal rat serum), blank groups (F-12K medium), and treatment groups containing 2%, 10%, 15%, or 20% raw or steamed PJR drug-containing sera. Cell proliferation was assessed using the CCK-8 assay (GLPBIO, Shanghai, China). A549 cells were seeded into 96-well plates at a density of 8 × 10^4^ cells/mL (100 μL/well) and incubated overnight at 37 °C. Each treatment condition was tested in triplicate. Cells were then treated with the designated concentrations of drug-containing serum for 24 h. Following treatment, 10 μL of CCK-8 solution was added to each well and incubated in the dark for 2 h. Absorbance was measured at 450 nm using a microplate reader. Cell viability was calculated according to the formula: Cell viability (%) = (OD_treated_ − OD_blank_)/(OD_control_ − OD_blank_) × 100%.

#### 4.7.6. Cell Morphology Observation

Log-phase A549 cells were seeded into 6-well plates at a density of 1 × 10^6^ cells/mL (1 mL/well) and incubated overnight at 37 °C with 5% CO_2_. Cells were then treated with 2%, 10%, 15%, or 20% drug-containing serum, along with a cell control group. After 24 h of incubation under the same conditions, cellular morphological changes were observed and photographed under an inverted microscope.

### 4.8. Statistical Analyses

Statistical analyses were performed using SPSS version 25.0. Data are expressed as mean ± standard deviation (SD). Intergroup differences were analyzed using one-way ANOVA, and *p* < 0.05 was considered statistically significant.

## 5. Conclusions

In this study, an integrated multi-method approach, combining metabolomics, network pharmacology, molecular docking, MD simulations, and experimental validation, was employed to elucidate the key constituents and mechanisms underlying the enhanced anti-lung cancer activity of steamed PJR. The results demonstrated that saponins such as Ginsenoside Rg3, Rg5, Rg6, Rh2, F1, F4, Vinaginsenoside R1, Momordin I and Ia, and (3β,21β)-12-oleanene-3,21,28-triol 28-[arabinosyl-(1→3)-arabinosyl-(1→3)-arabinoside] (Olean-12-ene-3β,21β,28-triol 28-O-arabinotrioside) constitute the major metabolites responsible for the therapeutic effects of steamed PJR. Five core targets, AKT1, EGFR, HSP90AA1, SRC, and STAT3, were identified as key mediators of its anti-lung cancer action, participating in critical signaling pathways including MAPK, PI3K-Akt, and Ras pathways. The enhanced anti-lung cancer efficacy of steamed PJR was further supported by in vitro experiments. Collectively, this work provides new insights into the chemical transformations induced by steaming and their contribution to the improved pharmacological activity of PJR, offering a scientific foundation for its potential development as an anti-lung cancer therapeutic or functional health product.

## Figures and Tables

**Table 1:** Differential metabolites of PJR before and after steaming.

Peak ID	RT (min)	Identification	Molecular Formula	Ions	Exact Mass	Error (ppm)	MS/MS Fragment	VIP	*p*	Trend
1	6.37	Notoginsenoside J	C42H74O16	[M−H]^−^	833.4899	1.47	671.4501, 509.4091, 453.1986	1.61	2.69 × 10^−2^	↓
2	9.79	Notoginsenoside R1	C47H80O18	[M−H]^−^	931.5267	2.80	799.5081, 637.45000, 475.3922	4.61	4.04 × 10^−5^	↓
3	10.9	Ginsenoside B2	C48H82O18	[M−H]^−^	945.5400	3.10	783.5142, 637.4500, 475.3922	1.33	4.91 × 10^−10^	↓
4	17.97	Notoginsenoside R4	C59H100O27	[M−H]^−^	1239.6345	3.25	807.4406, 645.3884, 569.3983	2.85	3.39 × 10^−8^	↓
5	19.31	Notoginsenoside Fa	C59H100O27	[M−H]^−^	1239.6345	2.68	879.5244, 623.3054, 593.2994	2.56	1.61 × 10^−4^	↓
6	20.99	Ginsenoside F1	C36H62O9	[M−H]^−^	637.4299	0.49	597.2381, 461.2545, 345.1388	7.21	1.89 × 10^−15^	↑
7	23.78	Vinaginsenoside R7	C53H90O22	[M−H]^−^	1077.5820	3.38	955.5255, 793.4662, 569.4081	1.35	1.90 × 10^−3^	↓
8	30.21	Ginsenoside Rd	C48H82O18	[M+HCOO]^−^	991.5454	3.16	793.4662, 725.4743, 419.2361	8.94	4.13 × 10^−6^	↓
9	30.34	Ginsenoside Rs2	C55H92O23	[M+HCOO]^−^	1165.5979	3.56	991.5887, 793.4662, 605.3209	1.8	1.90 × 10^−2^	↓
10	32.18	Ginsenoside Rs1	C55H92O23	[M+HCOO]^−^	1165.5979	3.16	605.3209, 592.2969	1.39	1.30 × 10^−2^	↓
11	32.45	Gypenoside XVII	C48H82O18	[M+HCOO]^−^	991.5454	2.72	945.5776, 869.5281, 518.2928	3.98	5.47 × 10^−4^	↓
12	33.22	Calenduloside H methyl ester	C49H78O19	[M+HCOO]^−^	1015.5091	2.92	853.4913, 530.2729, 455.3732	2.28	3.58 × 10^−6^	↓
13	33.23	Calenduloside G methyl ester	C43H68O14	[M+HCOO]^−^	853.4566	1.94	609.4003, 455.3688, 153.0221	1.22	1.84 × 10^−7^	↓
14	34.87	Ginsenoside F4/Rg6	C42H70O12	[M+HCOO]^−^	811.4824	0.39	583.3910, 537.3824, 409.1258	4.81	2.24 × 10^−14^	↑
15	40.54	Momordin I	C41H64O13	[M−H]^−^	763.4251	0.42	703.9182, 654.3607, 339.6309	1.46	4.98 × 10^−3^	↑
16	41.93	(3b, 21b)-12-Oleanene-3,21,28-triol 28-[arabinosyl-(1→3)-arabinosyl-(1→3)-arabinoside]	C45H74O15	[M−H]^−^	853.4929	1.75	793.4778, 731.4792, 455.3732	1.12	4.11 × 10^−12^	↑
17	42.12	Ginsenoside Rg3	C42H72O13	[M−H]^−^	783.4875	1.24	621.4654, 459.4040, 161.0519	2.62	4.41 × 10^−8^	↑
18	47.70	Vinaginsenoside R1	C44H74O15	[M−H]^−^	841.4929	1.84	795.5377, 455.3732, 279.2435	1.05	2.93 × 10^−4^	↑
19	48.41	Momordin Ia	C42H66O13	[M−H]^−^	777.4407	1.48	627.4192, 316.8525, 279.2469	1.12	2.78 × 10^−3^	↑
20	48.73	Ginsenoside Rg5	C41H68O10	[M−H]^−^	765.4770	0.69	603.4623, 501.3495, 113.0265	2.29	4.13 × 10^−6^	↑
21	50.75	Ginsenoside Rh2	C36H62O8	[M+HCOO]^−^	667.4404	0.81	569.3983393.2220, 146.9706	2.89	6.67 × 10^−6^	↑

Note: ↑ and ↓ indicate a significant increase and decrease.

## Data Availability

The original contributions presented in this study are included in the article/Supplementary Material. Further inquiries can be directed to the corresponding authors.

## Supplementary Materials

The following supporting information can be downloaded at https://www.mdpi.com/article/10.3390/ijms262411999/s1.

## Abbreviations

The following abbreviations are used in this manuscript:

PJR	*Panacis Japonici Rhizoma*
UPLC-Q-TOF-MS	Ultra-high performance liquid chromatography-quadrupole/time-of-flight mass spectrometry
TCM	Traditional Chinese medicine
PCA	Principal component analysis
OPLS-DA	Orthogonal partial least squares-discriminant analysis
VIP	Variable importance in projection
PPI	Protein–protein interaction
GO	Gene Ontology
KEGG	Kyoto Encyclopedia of Genes and Genomes
DEGs	Differentially expressed genes
CMC-Na	Carboxymethyl cellulose sodium
RMSD	Root mean square deviation
RMSF	Root mean square fluctuation
Rg	Radius of gyration
SASA	Solvent accessible surface area
